# Performance of Biomarkers FibroTest, ActiTest, SteatoTest, and NashTest in Patients with Severe Obesity: Meta Analysis of Individual Patient Data

**DOI:** 10.1371/journal.pone.0030325

**Published:** 2012-03-14

**Authors:** Thierry Poynard, Guillaume Lassailly, Emmanuel Diaz, Karine Clement, Robert Caïazzo, Joan Tordjman, Mona Munteanu, Hugo Perazzo, Bernard Demol, Robert Callafe, François Pattou, Frederic Charlotte, Pierre Bedossa, Philippe Mathurin, Vlad Ratziu, for the FLIP consortium

**Affiliations:** 1 APHP UPMC Liver Center, Paris, France; 2 Hepatology Department, Centre Hospitalier Lille, Lille, France; 3 Hepatology Department, Centre Hospitalier Bethune, Bethune, France; 4 Institute of Cardiometabolisme and Nutrition, APHP Pitié-Salpêtrière Hospital, Paris, France; 5 INSERM, U872, Nutriomique, Paris, France; 6 Université Pierre et Marie Curie-Paris 6, Centre de Recherche des Cordeliers, Paris, France; 7 Biopredictive, Paris, France; 8 Assistance Publique-Hôpitaux de Paris, Hôpital Beaujon, Département de Pathologie Clichy, Clichy, France; Copenhagen University Hospital Gentofte, Denmark

## Abstract

**Background:**

Liver biopsy is considered as the gold standard for assessing non-alcoholic fatty liver disease (NAFLD) histologic lesions in patients with severe obesity. The aim of this study was to perform an overview of 3 studies which assessed the performance of non-invasive markers of fibrosis (FibroTest), steatosis (SteatoTest) and steato-hepatitis (NashTest, ActiTest) in these patients.

**Methods:**

494 patients with interpretable biopsy and biomarkers using of three prospective cohorts of patients with severe obesity (BMI >35 kg/m2) were included. Histology (NAS score) and the biochemical measurements were blinded to any other characteristics. The area under the ROC curves (AUROC), sensitivity, specificity, positive and negative predictive values were assessed. Weighted AUROC (wAUROC Obuchowski method) was used to prevent multiple testing and spectrum effect. Two meta-analyses were performed; one used the individual patient, and the other a classical meta-analysis.

**Results:**

Prevalence of advanced fibrosis (bridging) was 9.9%, advanced steatosis (>33%) 54.2%, and steato-hepatitis (NAS score >4) 17.2%. The mean wAUROCs were: FibroTest for advanced fibrosis (95%CI; significance)  =  0.85 (0.83–0.87; P<0.0001); SteatoTest for advanced steatosis = 0.80 (0.79–0.83); and ActiTest for steato-hepatitis = 0.84 (0.82–0.86; P<0.0001). Using the classical meta-analysis (random effect model) the mean AUROCs were: FibroTest = 0.72 (0.63–0.79; P<0.0001); SteatoTest = 0.71 (0.66–0.75; P<0.0001); and ActiTest = 0.74 (0.68–0.79; P<0.0001). Despite more metabolic risk factors in one cohort, results were similar according to gender, presence of diabetes and between the 3 cohorts.

**Conclusion:**

In patients with severe obesity, a significant diagnostic performance of FibroTest, SteatoTest and ActiTest was observed for liver lesions.

## Introduction

Severe obesity is associated with decreased life expectancy [1]. In terms of liver injury, severe obesity is implicated in development of non- alcoholic fatty liver disease (NAFLD), including steatosis, non alcoholic steato-hepatitis (NASH) and fibrosis [2]–[4].

Non invasive biomarkers of liver fibrosis have been extensively validated in chronic viral hepatitis and more recently in patients with alcoholic and non alcoholic fatty liver diseases, the most validated serum fibrosis biomarkers being FibroTest®/FibroSure® (FT) [5]–[8]. In patients at high risk of NAFLD, FT has been validated in two studies [9], [10].

Very few biomarkers have been validated for the diagnosis of steatosis or NASH, including patients with severe obesity [7], [8], [10].

The aim of the present study was to better assess the performance of 4 previously published biomarkers, of fibrosis (FT) [9], of steatosis (SteatoTest®)(ST) [11] and of necrosis and inflammation [ActiTest® (AT) and NashTest® (NT)] [12], the combination of these 4 biomarkers is named FibroMax®, in patients with severe obesity. As part of the FLIP consortium European project [13], a large integrated database of 494 patients was constructed using 3 recent validation studies performed independently from the inventor's group permitting to increase of number of patients with advanced liver injuries.

The specific goal was to estimate the diagnostic performance of these biomarkers versus ALT the routine liver test, using the most accurate methods already applied in patients with chronic hepatitis C: meta-analysis of individual data [14], and standardized area under the characteristics receiver operating curves (AUROCs) [15]–[17].

## Methods

Informed consent have been obtained for all patients and all clinical investigation have been conducted according to the principles expressed in the Declaration of Helsinki. The ethic committee of Groupe Hospitalier Pitié Salpêtrière has approved the research.

We identify all clinical studies assessing the diagnostic performance of FT, ST, AT and NT, in obese patients. Two meta-analyses were performed. One used the integrated database of these studies combining individual data provided by authors, and the other was a classical meta-analysis of these studies but using weighted AUROCs. Finally the performances of the four biomarkers and ALT were assessed using methods without gold standard.

### Patients

To be eligible for the study, all patients had to have fulfilled the following criteria: (1) severe obesity (BMI>35 kg/m2), (2) absence of current excessive drinking, as defined by average daily consumption of alcohol of 20 g/day for women and 30 g/day for men; (3) absence of long-term consumption of hepatotoxic drugs; and (4) negative screening for chronic liver diseases, including negative testing for hepatitis B surface antigen and hepatitis C virus antibodies, and no evidence of genetic hemochromatosis.

The following clinical and biological features were required for the integrated data base: weight, height, BMI, blood pressure, alanine aminotransferase (ALT), gamma glutamyl transferase (GGT), serum triglyceride, cholesterolemia, fasting blood glucose and interpretable biomarkers FT, ST, AT and NT. Diabetes, hypercholesterolemia and hypertriglyceridemia were defined as follows: fasting blood glucose>1.26 g/l, cholesterolemia>2.4 g/l and serum triglyceride>1.5 g/l, or respective specific treatment.

### Biomarkers measurements

FT, ST, AT and NT (Biopredictive, Paris, France; Fibro-SURE® is the brand name for FT in USA, LabCorp, Burlington, NC, USA) were determined as has been previously published [6], [14]. The published recommended pre-analytical and analytical procedures were used [6], [14]. FT includes α2-macroglobulin, apolipoprotein A1, haptoglobin, total bilirubin, and GGT, adjusted for age and gender; AT includes same 5 components plus transaminases ALT; ST and NT included the same 6 components than AT plus serum glucose, triglycerides and cholesterol, adjusted for age, gender and BMI.

FT, AT and ST scores range from zero to 1.00, with higher scores indicating a greater probability of significant lesions. The predetermined FT conversion for the METAVIR fibrosis stage scoring system is 0.00–0.27 for F0; >0.27–0.48 for F1; >0.48–0.58 for F2; >0.58–0.74 for F3; >0.74 for F4 [14]. The predetermined AT conversion for the METAVIR activity grade scoring system is 0.00–0.17 for A0; >0.17–0.52 for A1; >0.52–0.62 for A2; >0.62 for A3 [17]. The predetermined ST conversion for steatosis grade is 0.00–0.57 for S0; >0.57–0.69 for S1; and >0.69–1.000 for S2–S3 [11]. The NT is a 3 categories score for predicting 3 NAS categories: 0.25 is “No-Nash”, 0.50 “Possible Nash” and 0.75 “Nash” [12].

Patents reference were for FT-AT: USPTO #6631330, ST #20090111132 and NT: #20080145864. Standard manufacturer algorithms were used to exclude high risk profile of false negative/positive [6], [14], [18]–[19].

### Histological analysis

In the three studies, histological features were scored according to the same criteria than those used in the FT/AT [9], [17], ST [11], and NT [12] validations in non-alcoholic fatty liver disease (NAFLD), and those used in the NAFLD scoring system (NAS) [20]. Fibrosis was scored using a predetermined scoring system equivalent to METAVIR scoring system [20]–[22] and used in the first FT validation in NAFLD [9]. Fibrosis was staged on a scale of 0 to 4: F0 – no fibrosis; F1 – portal fibrosis or perivenular fibrosis without septa; F2 – few septa; F3 – numerous septa without cirrhosis; and F4 – cirrhosis.

Steatosis was quantified by low- to medium-power evaluation of parenchymal involvement by steatosis (percentage of steatosis). Steatosis was scored using the NAS scoring system from 0 to 3 with a four grades scoring system from S0 to S3: S0–no steatosis or <5%; S1–5% to 33%; S2- >33% to 66% and S3 >66% [11], [20].

NASH was classified using the NAS score [20], defined as the sum of scores for steatosis (0–3), lobular inflammation (0–3) and ballooning (0–2), thus ranging from 0 to 8. Cases with NAS of 0 to 2 were considered not diagnostic of NASH; cases with scores of 5 or greater were diagnosed as NASH. Cases with activity scores of 3 and 4 were considered as borderline, possible NASH [20]. In each population, liver biopsies were classified by a centralized pathologists blinded to the clinical and biological data. Liver biopsies were performed during the operative procedure, by Hepafix needle in half of cases. Patients with more than 6 months between biopsy and serum samples were not included. Biopsies were routinely stained with hematoxylin-eosin and Masson's trichrome.

### Statistical analysis

Methods were detailed in **File S1 and Figure S1**. In order to take into account the spectrum effect and to prevent multiple testing risk, the primary endpoint for each quantitative biomarker's performance (FT, AT, ST) was the Obuchowski measure [14]–[16]. This measure is a multinomial version of the AUROC. With N categories of the gold standard outcome (i.e. histological fibrosis stage) and AUROCst, the estimate of the AUROC of diagnostic tests for differentiating between categories s and t, the Obuchowski measure, is a weighted average of the N(N−1)/2 different AUROCst corresponding to all the pairwise comparisons between 2 of the N categories. Each pairwise comparison between stages has been weighted (wAUROC) to take into account the distance between grades or stages. AMSTAR recommendations were followed for the meta-analysis [23]. The secondary outcomes were the AUROC using the standard definition of liver injury and predictive values using predetermined cutoffs as defined in the validation of biomarkers in NAFLD [6], [9], [10], [11], [12], [14], [17], [20]. A sensitivity analysis of biomarkers analysis was performed in patients with diabetes versus patients without diabetes, according to gender and according to age (50 years cutoff).

## Results

### Studies search

A total of 212 studies of biomarkers have been identified in patients with obesity or NAFLD, including 90 studies of steatosis' biomarkers, 54 studies of fibrosis' biomarkers, and 51 of steato-hepatitis' biomarkers.

**Figure 1:**
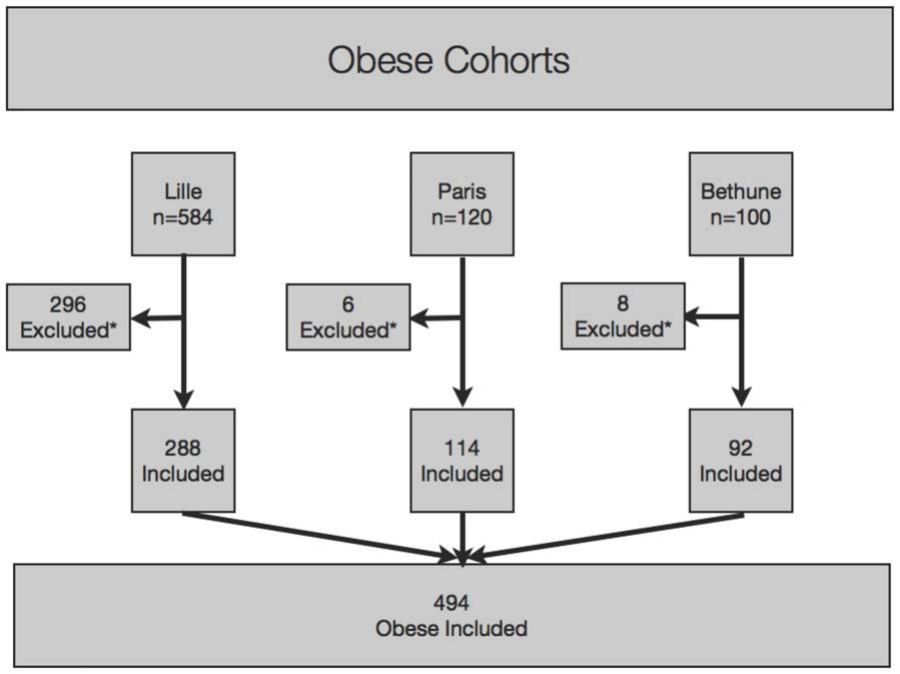
Included patients with morbid obesity. *In the Lille cohort a total of 296 patients have been excluded, 94 because histological staging was missing, 19 were duplicate, 229 for biomarkers non assessed (including 39 with more than one cause of exclusion) and 3 patients with not interpretable FT/ST (extreme value of triglycerides/glucose/ApoA1 detected by security algorithms). In the Paris cohort 6 patients have excluded, 2 with BMI lower than 35, 3 because histological staging was missing, and one for biomarkers not assessed. In the Bethune cohort 8 patients have excluded, 3 with BMI lower than 35, and 5 because histological staging was missing.

Among these 212 studies, three were included as specifically conducted in patients with severe obesity (Figure 1 and **Figure S2**): three [10], [24], [25] assessed FT, ST, AT and NT. One study is part of an ongoing cohort (Lille cohort) [10], [26]; for the other two (Paris and Bethune cohorts) [24], [25] the performances of biomarkers were not detailed in the publications but the authors shared the individual data; five other studies investigated these tests in patients with NAFLD but not specifically in severe obese patients and were not included: FT [9], [13], ST [11], [13], NT 12,13.

### Patients included (Figure 1)

In the Lille cohort, 288 patients were included [10], [26], 114 in the Paris cohort, and 84 in the Bethune cohort. Between the cohorts there was few significant differences, mostly less metabolic factors in the Bethune cohort (Table 1). There was no significant difference between included and non included patients' characteristics.

**Table 1:** Characteristics of 494 obese patients included in each population.

Characteristics	Lille n = 288	Paris n = 114	Bethune n = 92	All n = 494
Female sex: No (%)	220 (76.4%)	89 (78.1%)	73 (79.4%)	382 (77.3%)
Age (years): mean (SD)	41.6 (12.8)	43.7 (11.3)	43.6 (10.1)	42.2 (11.3)
BMI (kg/m2): mean (SD)	48.6 (8.9)	46.5 (7.6)	45.1 (6.1)	47.4 (7.9)
Diabetes mellitus: No (%)	92 (31.9%)	38 (33.3%)	11 (12.0%)	141 (28.5%)
Arterial hypertension : No (%)	174 (60.4%)	11 (61.1%) out of 18^1^	32 (34.8%)	217 (54.5%) out of 398^1^
Dyslipidemia No (%)	167 (58.0%)	43 (46.2%) out of 93^1^	18 (19.6%)	228 (48.2%) out of 473^1^
Cholesterolemia (g/l): mean (SD)	4.94 (0.88)	5.01 (0.98)	5.81 (1.07)	5.11 (1.00)
Serum triglycerides (g/l): mean (SD)	1.58 (0.88)	1.61 (0.97)	1.62 (0.75)	1.60 (0.88)
ALT (IU/l): mean (SD)	34 (23)	42 (33)	35 (26)	36 (26.5)
GGT (IU/l): mean (SD)	44 (48)	58 (98)	45 (85)	47 (70)
Fasting blood glucose (g/l): mean (SD)	6.2 (2.4)	6.1 (2.7)	5.5 (2.0)	6.0 (2.2)
Biopsy length (mm) mean (SD)	13.6 (11.0) out of 262^1^	16.3 (7.1) out of 21^1^	not available	13.8 (10.8) out of 283^1^
Fibrosis F0/F1/F2/F3/F4: No (%) (METAVIR scoring system)	170 (59.0%)/98 (34.0%)/13 (4.5%)/2 (0.7%)/5 (1.7%)	48 (42.1%)/43 (37.7%)/16 (14.0%)/5 (4.4%)/2 (1.8%)	22 (23.9%)/64 (69.6%)/0 (0%)/5 (5.4%)/1(1.1%)	240 (48.6%)/205 (41.5%)/29 (5.8%)/12 (2.4%)/8 (1.6%)
Inflammation I0/I1/I2/I3 (Kleiner score)	196 (71.3%)/64 (23.3%)/11 (4.0%)/4 (1.5%) out of 275^1^	48 (42.1%)/60 (52.6%)/6 (5.3%)	57 (62.0%)/32 (34.8%)/3 (3.3%)	301 (62.6%)/156 (32.4%)/20 (4.2%)/4 (0.8%) out of 481^1^
Ballooning B0/B1/B2: No (%) (Kleiner score)	237 (86.2%)/24 (8.7%)/14 (5.1%) out of 275^1^	41 (36.0%)/44 (38.6%)/29 (24.4%)	27 (29.4%)/43 (46.7%)/22 (23.9%)	282 (57.1%)/127 (25.7%)/85 (17.2%) out of 481^1^
Steatosis S0/S1/S2–S3 (Kleiner score)	36 (12.5%)/113 (39.2%)/139 (48.3%)	20 (17.5%)/32 (28.1%)/62 (54.4%)	13 (14.1%)/29 (31.5%)/50 (54.4%)	69 (14.0%)/157 (31.8%)/268 (54.2%)
Extent of steatosis (%): mean (SD)	31.2 (24.9)	41.7 (33.7)	40.9 (28.3)	35.4 (28.2)
NAS score (Kleiner)				
0–2 No Nash	203 (72.6%)	40 (35.1%)	39 (42.4%)	282 (57.1%)
3–4 Possible	63 (20.4%)	34 (29.8%)	30 (32.6%)	127 (25.7%)
5–8 Nash	22 (6.9%)	40 (35.1%)	23 (25.0%)	85 (17.2%)

1 When data are missing the number of patients with data not missing is given.

### Integrated analysis

The AUROCs were detailed in **Figure S2**.

#### Performance of FibroTest for the diagnosis of fibrosis

Prevalence of advanced fibrosis (METAVIR stage F2-F3-F4) was 9.9% (Table 1).

The FT mean wAUROC was (95%CI; significance vs random) 0.85 (0.83–0.87; P<0.0001) and for ALT 0.84 (0.82–0.86; P<0.0001 not different vs FT; Z = 0.3; P = 0.77). Pairwise comparisons between stages are given in table 2. FT wAUROC was also highly significant in 141 patients with diabetes 0.80 (0.78–0.82; P<0.0001).

**Table 2:** Accuracy (weighted area under the ROC curves) of FibroTest, SteatoTest, ActiTest and ALT for the diagnosis of fibrosis, steatosis and NASH adjacent stages/grades in 494 patients with morbid obesity.

	Fibrosis			
	F1 (n = 205) vs F0 (n = 240)	F2/F3/F4 (n = 49) vs F1 (n = 205)	F2/F3/F4 (n = 49) vs F0 (n = 205)	Obuchowski measure (n = 494)
FibroTest	0.532 (0.011)	0.545 (0.039)	0.651 (0.034)	0.837 (0.005)
ALT	0.546 (0.017)	0.508 (0.040)	0.626 (0.036)	0.839 (0.006)

	Steatosis			
	S1 (n = 157) vs S0 (n = 69)	S2/S3 (n = 268) vs S1 (n = 157)	S2/S3 (n = 268) vs S0 (n = 69)	
SteatoTest	0.586 (0.040)	0.532 (0.025)	0.777 (0.028)	0.803 (0.011)
ALT	0.525 (0.017)	0.539 (0.018)	0.601 (0.018)	0.746 (0.006)

	Nash			
	Possible (n = 127) vs No (n = 282)	NASH (n = 85) vs Possible (n = 127)	NASH (n = 85) vs No (n = 282)	
ActiTest	0.702 (0.026)	0.549 (0.031)	0.682 (0.029)	0.839 (0.009)
ALT	0.572 (0.021)	0.531 (0.032)	0.613 (0.027)	0.806 (0.007)

Note that the overall Obuchowski measure is not equivalent to an usual area under the ROC curve as weighted according to the distance between stages/grades.

The overall mean (SE) accuracy of FibroTest (Obuchowski measure) was not significantly greater than that of ALT, Z = −0.3 P = 0.77.

The overall accuracy of SteatoTest was significantly greater than that of ALT, Z = 5.2 P<0.0001.

The overall mean accuracy of Actitest (Obuchowski measure) was significantly greater than that of ALT, Z = 4.6 P<0.0001.

**Figure 2:**
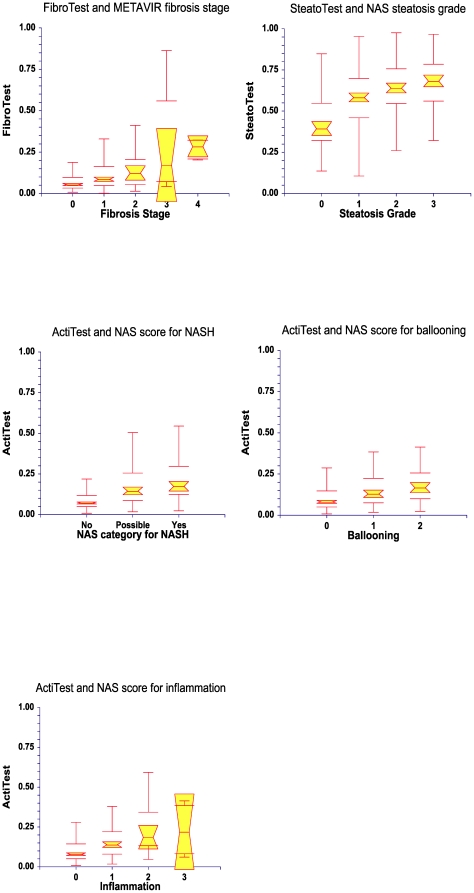
Box Plots of Biomarkers according to liver injury. FibroTest according to fibrosis stage (Panel A), SteatoTest according to steatosis grade (Panel B), ActiTest according to NAS score (Panel C) to ballooning (Panel D) and inflammation (Panel E) in 494 patients. Notched box plots showing the relationship between tests and the stage/grade of liver injury. The horizontal line inside each box represents the median, and the width of each box the median±1.57 interquartile range/√n (to assess the 95% level of significance between group medians). Failure of the shaded boxes to overlap signifies statistical significance (P<0.05). The horizontal lines above and below each box encompass the interquartile range (from the 25th to 75th percentile), and the vertical lines from the ends of the box encompass the adjacent values (upper: 75th percentile plus 1.5 times interquartile range; lower: 25th percentile minus 1.5 times interquartile range).

Classical AUROC of FT was 0.72 (0.63–0.79; P<0.0001). The FT values according to each stage are given in Figure 2A.

#### Performance of SteatoTest for the diagnosis of steatosis

Prevalence of advanced steatosis (>33%) was 54.2% (Table 1). Severe steatosis (>66%) represented 20.2% (100/494) of patients versus 34% (168/494) for marked steatosis (33–66%). The ST mean weighted AUROC was 0.80 (0.79–0.83) significantly greater (Z = 5.2 P<0.0001) than that of ALT 0.75 (0.73–0.77; P<0.0001). Pairwise comparisons between steatosis grades (S0/S1/S2S3) are given in Table 2. ST weighted accuracy was also highly significant in 141 patients with diabetes 0.76 (0.72–0.80; P<0.0001). Classical AUROC of ST was 0.71 (0.66–0.75; P<0.0001). The ST values according to each steatosis grade are given in Figure 2B.

#### Performance of NashTest, and ActiTest for the diagnosis of NASH

Prevalence of NASH was 17.2% and for possible NASH 25.7% (Table 1). Concordance rate between histological NAS score and presumed by NASH test was 33.1% (P<0.0001) but with a weak kappa reliability test  =  0.18. Among 110 patients presumed No-Nash by NT, 95 (86%) were No-Nash, 10 Possible and 5 Nash at biopsy; among 355 presumed Possible-Nash by NT, 176 were No-Nash, 111 (31%) Possible and 68 Nash at biopsy; among 29 patients presumed Nash by NT, 11 were No-Nash, 6 Possible and 12 (41%) Nash at biopsy.

Using quantitative biomarker AT, the wAUROC was highly significant for the diagnosis of Nash = 0.84 (0.82–0.86; P<0.0001) significantly greater (Z = 4.6 P<0.0001) than that of ALT, 0.81 (0.80–0.82; P = 0.007).

Pairwise comparisons between classes (NoNash/Possible/Nash) are given in table 2. AT wAUROC was also highly significant in 141 patients with diabetes 0.81 (0.78–0.84; P<0.0001). Classical AUROC of AT was 0.74 (0.68–0.79; P<0.0001). The AT values according to each NAS classes are given in Figure 2C.

#### Sensitivity, specificity, positive (PPV) and negative(NPV) predictive values

Diagnostic values according to predetermined cutoffs are detailed in Table 3. For fibrosis the PPV was 87.5% for the diagnosis of Fibrosis >F0 using the 0.27 cutoff and the NPV for fibrosis >F1 was 93.8% using 0.48 cutoff.

**Table 3:** Sensitivity, specificity and predictive values of biomarkers according to predetermined cutoffs in 494 patients with morbid obesity.

Biomarker (cutoff)	Disease (Prevalence)	Se	NPV	Sp	PPV
FibroTest (0.27)	>F0 (51.4%)	34/118 13.4%^1^	234/454 51.5%	234/240 97.5%	34/40 85.0%
FibroTest (0.48)	>F1 (9.9%)	4/49 8.2%	443/488 90.8%	443/445 99.6%	4/6 66.7%
SteatoTest (0.38)	>S0 (86.0%)	381/425 89.7%	31/75 41.3%	31/69 44.9%	381/419 90.9%
SteatoTest (0.69)	>S1 33% (54.3%)	103/268 38.4%	184/349 52.7%	184/226 81.4%	103/145 71.0%
NashTest (0.70)	NAS>4 (17.2%)	12/85 14.1%	392/465 84.3%	392/409 95.8%	12/29 41.4%
NashTest (0.50)	NAS>2 (42.9%)	197/212 92.9%	95/110 86.4%	95/282 33.7%	197/384 51.3%
ActiTest (0.29)	NAS>4 (17.2%)	24/85 28.2%	371/432 85.9%	371/409 90.7%	24/62 38.7%
ActiTest (0.17)	NAS>2 (42.9%)	92/212 43.4%	241/361 66.8%	241/282 85.5%	92/133 69.2%

1 number of patients n/N and %.

For steatosis the PPV of ST was 92.4% for the diagnosis of steatosis >S0 using the 0.38 cutoff and the NPV for steatosis >S1 was 59.3% using 0.69 cutoff.

For steato-hepatitis the NPV of AT was 96.0% for the diagnosis of NASH (NAS>4) using the 0.29 cutoff and the PPV for Possible/NASH or NASH (NAS>2) was 47.5% using 0.17 cutoff.

Sensitivity analysis of AUROCs (**Table S1**) and Obuchowski measures (**Table S2**) according to the presence of diabetes, gender and age showed that the performances of FT, AT and ST remained always highly significant for the diagnosis of advanced fibrosis by FT and NASH by AT. For ST the AUROC was significantly higher in patients with diabetes than without.

### Meta-analysis of the 3 studies

**Figure 3:**
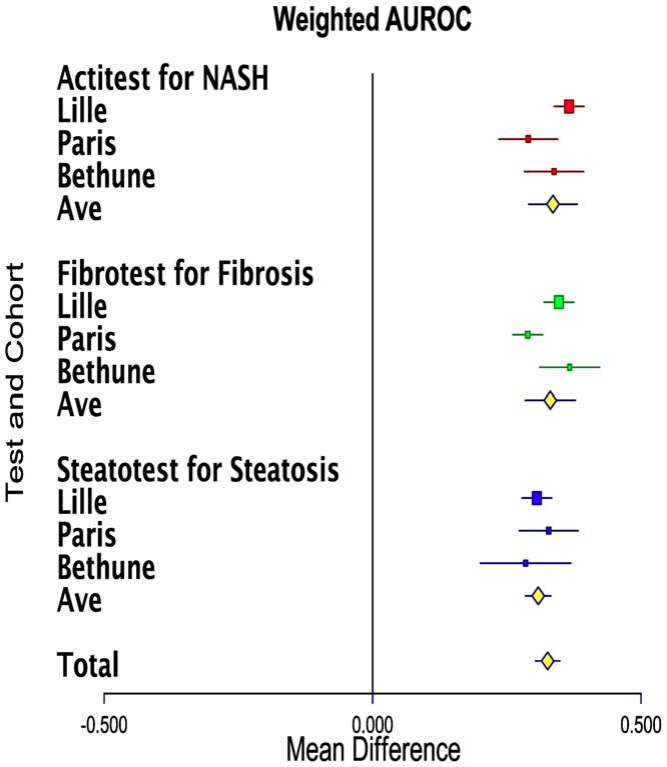
Meta-analyses of 3 studies of biomarkers accuracy (Obuchowski measure: weighted area under the ROC curve [wAUROC]) for the diagnostic of liver injury in patients with severe obesity. FibroTest for the diagnosis of advanced fibrosis (at least equivalent to METAVIR score F2). SteatoTest for the diagnosis of advanced steatosis (>30% steatosis). ActiTest for the diagnosis of NASH (NAS score >4). The horizontal lines indicate the 95% confidence interval for the mean difference between between test's wAUROCs and random (0.500). The vertical lines indicate the equivalence line (0% difference). Positive differences indicate a difference in favor of test). When the horizontal line crosses the vertical line, there is no significant difference. Ave = Average of AUROCs.

The pooled results of wAUROCs are shown in Figure 3 for FT and advanced fibrosis, for ST and advanced steatosis and for AT and NASH. For FT the mean wAUROC was 0.83 (0.78–0.88; P<0.0001), with a significant heterogeneity (Q = 11.1 P = 0.004). For ST the mean wAUROC was 0.81 (0.78–0.83; P<0.0001), without significant heterogeneity (Q = 0.8 P = 0.67). For AT the mean wAUROC was 0.84 (0.79–0.88; P<0.001), without significant heterogeneity (Q = 5.9 P = 0.051).

## Discussion

This study is the largest analysis of liver biomarkers (FT, ST, AT and NT) performances in patients with severe obesity. This overview confirms the accuracy previously observed for the diagnosis of liver injury in patients with NAFLD [9], [11], [12] and in general populations [27], [28]. The two new studies (Paris and Bethune' cohorts) performed in patients with severe obesity have confirmed the performances previously observed in the Lille [10].

### Advantages of this overview

The main advantage of this overview was an increase of power in comparison with isolated studies. A large number of patients was necessary to assess correctly these biomarkers performances as some classes of liver injury could be too small, such as patients with advanced fibrosis or patients without advanced steatosis. Indeed the liver injury spectrum is dramatically different in obese patients than in liver diseases where FT and ST were originally constructed. In obese we observed 9.9% of advanced fibrosis, much lower than the 49.0% observed in patients with chronic hepatitis C [29]; the prevalence of steatosis (>5%) was 85.9% much higher than the 45.0% of the initial ST training group [11].

Due to this spectrum effect the use of the Obuchowski measure was also necessary to prevent misleading interpretation of not weighted AUROCs [14]–[16]. With or without standardization, the AUROCs of FT was 0.85 vs 0.72, ST was 0.80 vs 0.71 and AT was 0.84 vs 0.74, respectively.

The accuracy of the biomarkers were confirmed using several statistical methods the integrated data base analysis and the classical meta-analysis. There was no difference between cohorts and between patients with or without diabetes.

### Limitation of the present study

This population of tertiary centers offering bariatric surgery is not representative of the general population of severe obese patients. There was an heterogeneity between the three cohorts with less metabolic factors in the Betune cohort. The distribution of the present study sample was taken for he Obuchowski measure as the present study was the largest study published in severe obese and there was no recognized reference distribution. Due to the limited number of patients with advanced fibrosis it was therefore not possible in the present study to compare the accuracy between all advanced fibrosis stages. Only 8 (1.6%) patients had a cirrhosis. This low prevalence of advanced fibrosis was expected as these obese patients were selected according to the absence of other recognized risk factors of fibrosis progression: no high alcohol consumption, predominantly young (42 years old) and females (77.3%) [21].

ST has limitations as it is mostly a semi quantitative test mostly designed to be sensitive for excluding steatosis and it cannot not discriminate severe steatosis (greater than 66%) versus marked steatosis between 33% to 66%. More quantitative ST should be developed as severe steatosis represented 20% of these obese patients versus 34% for marked steatosis (33–66%).

This overview focused on 4 tests developed by several co-authors of the article, who have an obvious conflict of interest as inventor or employee of the company marketing these tests. However the other co-authors were totally independent, recruited the patients and performed the assay independently of the company and had a full access to all data and analyses.

Another limitation was the absence of direct comparisons with other biomarkers such as ELF, Fibrospect, Fibrometer and Fibroscan for fibrosis, cytokeratin 18 for NASH, and magnetic resonance imaging and spectroscopy for steatosis [7], [8], [30]. The main goal of this study was to validate the performance of these tests versus random. At least this overview demonstrated that both ST and AT were significantly more accurate than ALT for the diagnosis of steatosis and NASH in patients with severe obesity. There was no difference in the present study between the FT performance and the ALT performance for the diagnosis of fibrosis. This absence of significant difference should not be interpreted as an absence of difference according to the low power of this comparison. Due to the low prevalence of advanced fibrosis (9.9%) in obese patients a study comparing FT to other fibrosis biomarkers would need much more patients. As observed in other frequent liver disease, ALT is specifically associated with necro-inflammatory activity grades and therefore must not be used as a marker of fibrosis [17]. Ideally fibrosis' biomarkers must be interpreted together with validated independent biomarker of activity and steatosis to prevent false positive. Biomarker such as Fibrometer which included transaminases in its components, had a variability related to activity.

This overview confirms the significant accuracy of AT for the diagnosis of overt NASH as well as for the pairwise comparison between NAS categories, observed by Lassailly et al [10]. The AT was originally designed for necroinflammatory histological activity diagnosis in chronic hepatitis C and B. According to the observed performance in obese patients, it will be interesting to check the AT performance in patients with other NAFLD risk factors as well as a comparison or combination of cytokeratin 18 for the diagnosis of NASH.

Long term prospective studies must be undertaken in patients with severe obesity and other NAFLD risk factors in order to validate these biomarkers versus biopsy. In patients with chronic hepatitis C [31], chronic hepatitis B [32] and alcoholic liver disease [33] FT had similar the prognostic values than biopsy.

Finally a major limitation of liver biomarkers validation is the absence of perfect gold standard [19], [22]. Using more appropriate methodology such as latent class analysis looking for truth in the absence of gold standard is probably one scientific manner to better estimate the performance of liver biomarkers [34].

### Conclusion

In conclusion, as in patients with chronic hepatitis C, B and alcoholic liver disease, a significant diagnostic performance of FibroTest, SteatoTest and ActiTest was observed for liver lesions in patients with severe obesity.

## Supporting Information

Figure S1 **Prisma Flow diagram.**
(DOCX)

Figure S2 **Receiver Operating Characteristics (ROC) curves of biomarkers for the diagnosis of liver injury in patients with morbid obesity.** Figure S2A: Advanced fibrosis defined as METAVIR F2F3F4 (few septa, many septa, and cirrhosis). Mean non-weighted FibroTest AUROC (95%CI; significance vs random) = 0.72 (0.63–0.79; P<0.0001). Figure S2B: Advanced steatosis defined as steatosis = S2S3 (percentage steatosis > = 33%). Mean (not weighted) AUROC of SteatoTest was 0.71 (0.66–0.75; P<0.0001). Figure S3C: Nash defined as NAS score >4 (NASH). Mean (not weighted) AUROC of ActiTest was 0.74 (0.68–0.79; P<0.0001).
(DOCX)

Table S1 **Sensitivity analysis of AUROCs.**
(DOCX)

Table S2 **Obuchowski measures.**
(DOCX)

File S1 **Statistical methods.**
(DOCX)
