# Supplementary material for: Performance of Biomarkers FibroTest, ActiTest, SteatoTest, and NashTest in Patients with Severe Obesity: Meta Analysis of Individual Patient Data
Source: PLoS One. 2012 Mar 14;7(3):e30325. doi: 10.1371/journal.pone.0030325 (PMC3303768; doi:10.1371/journal.pone.0030325)
Supplement: Table S1 — Sensitivity analysis of AUROCs. (DOCX) [file pone.0030325.s003.docx]

**Supporting Information Table S1: Sensitivity analysis: Performance of FibroTest, SteatoTest, ActiTest and ALT for the diagnosis of advanced fibrosis, advanced steatosis and NASH in 494 patients with morbid obesity, according to presence of diabetes, gender and age (50 year cutoff); mean (se).**

|  | **AUROC FibroTest*** | **AUROC SteatoTest*** | **AUROC ActiTest*** |
| --- | --- | --- | --- |
| Characteristic (n) | F234 | S23 | A23 |
| Male (112) | 0.71 (0.07) | 0.60 (0.06) | 0.70 (0.05) |
| Female (382) | 0.69 (0.06 | 0.71 (0.03) | 0.77 (0.02) |
| Age<50 (365) | 0.72 (0.05) | 0.71 (0.03) | 0.79 (0.02) |
| Age>= 50 y (129) | 0.74 (0.07) | 0.65 (0.05) | 0.69 (0.05) |
| No diabetes (353) | 0.78 (0.06) | 0.72 (0.03)** | 0.78 (0.03) |
| Diabetes (141) | 0.65 (0.06) | 0.59 (0.05) | 0.72 (0.05) |

* All AUROCs highly significant vs random (P<0.01) ** P=0.01 between no-diabetes and diabetes
