# Supplementary material for: Performance of Biomarkers FibroTest, ActiTest, SteatoTest, and NashTest in Patients with Severe Obesity: Meta Analysis of Individual Patient Data
Source: PLoS One. 2012 Mar 14;7(3):e30325. doi: 10.1371/journal.pone.0030325 (PMC3303768; doi:10.1371/journal.pone.0030325)
Supplement: Table S2 — Obuchowski measures. (DOCX) [file pone.0030325.s004.docx]

**Supporting Information** **Table S2: Sensitivity analysis: Performance of FibroTest, SteatoTest, ActiTest and ALT for the diagnosis of advanced fibrosis, advanced steatosis and NASH in 494 patients with morbid obesity, according to presence of diabetes, gender and age (50 year cutoff); Obuchowski measure (OBU), mean (se).**

|  | **Obu FibroTest*** | **Obu SteatoTest*** | **Obu ActiTest*** |
| --- | --- | --- | --- |
| Characteristic (n) |  |  |  |
| Male (112) | 0.817 (0.012) | 0.796 (0.026) | 0.840 (0.018) |
| Female (382) | 0.841 (0.005) | 0.793 (0.011) | 0.826 (0.010) |
| Age<50 (365) | 0.829 (0.004) | 0.801 (0.011) | 0.843 (0.010) |
| Age>= 50 y (129) | 0.853 (0.012) | 0.782 (0.024) | 0.809 (0.016) |
| No diabetes (353) | 0.853 (0.005) | 0.797 (0.011) | 0.847 (0.011) |
| Diabetes (141) | 0.802 (0.011) | 0.765 (0.024) | 0.802 (0.016) |

* All measures highly significant vs random (P<0.001)
