# Supplementary material for: Performance of Biomarkers FibroTest, ActiTest, SteatoTest, and NashTest in Patients with Severe Obesity: Meta Analysis of Individual Patient Data
Source: PLoS One. 2012 Mar 14;7(3):e30325. doi: 10.1371/journal.pone.0030325 (PMC3303768; doi:10.1371/journal.pone.0030325)
Supplement: File S1 — Statistical methods. (DOCX) [file pone.0030325.s005.docx]

**Supporting Information File S1: Statistical methods**

***Obuchowski measure***

In order to take into account the spectrum effect and to prevent multiple testing risk, the primary endpoint for each quantitative biomarker's performance (FT, AT, ST) was the Obuchowski measure. This measure is a multinomial version of the AUROC. With N categories of the gold standard outcome (histological fibrosis stage or activity grade) and AUROCst, the estimate of the AUROC of diagnostic tests for differentiating between categories s and t, the Obuchowski measure, is a weighted average of the N(N –1)/2 different AUROCst corresponding to all the pairwise comparisons between 2 of the N categories. Each pairwise comparison has been weighted to take into account the distance between grades or stages (i.e., the number of units on the ordinal scale). A penalty function proportional to the difference (distance), in METAVIR or steatosis scores, between stages or grades was defined. For fibrosis, due to the small prevalence of stages F2, F3 and F4, 3 classes were compared: F0, F1, and F2F3F4; the penalty function was 0.25 between F0 and F1, 0.50 between F1 and F2-F3-F4 and 0.75 between F0 and F2-F3-F4. For activity (4 classes) the penalty function was 0.33 when the difference between grades was 1, 0.67 when the difference was 2, and 1 when the difference was 3. For steatosis (3 classes S0, S1, S2-S3 the penalty function was 0.25 between S0 and S1, 0.50 between S1 and S2-S3 and 0.75 between S0 and S2-S3. The Obuchowski measure can be interpreted as the probability that the noninvasive index will correctly rank 2 randomly chosen patient samples from different stages/grades according to the weighting scheme, with a penalty for misclassifying patients. Weighting can be based on the relative proportion of the stages/grades in the study sample, or on a reference distribution of stages/grades. The distribution of the present study sample was taken as this is the largest study published in severe obese and there was no recognized reference distribution. Note that the overall Obuchowski measure is not equivalent to an usual area under the ROC curve as the measure is the mean of all pairwise AUROCs weighted according to the distance between stages/grades or to the relative proportion of stages/grades.

***Meta-analysis***

**AMSTAR recommendations were followed for the meta-analysis. 1)** The research question (performance of 4 biomarkers) and inclusion criteria (severe obese patients with biopsy) have been be established before the conduct of the review. 2) TP and MM were two independent data extractors and there a third expert (VR) was scheduled in case of disagreements. 3) **A comprehensive literature search was performed using** MEDLINE and SCOPUS between January 2002 and August 2010 with the following key words combined: Obesity, NAFLD, Biomarkers (Fibrosis, Steatosis, Hepatitis) FibroTest, FibroSure, ActiTest, SteatoTest, and NashTest; the search was supplemented by consulting reviews, abstracts books of European, American and Asian Liver societies, contacting experts in the field and by reviewing the references in the studies found. 4) The search included reports regardless of their publication type. We excluded reports published as abstracts. 5) The list of studies included and excluded were provided. 6) The **characteristics of the included studies were provided. 7) Only studies with validated biomarkers and validated histological scoring system were included. 8) The quality of included studies were used appropriately in formulating conclusions. 9) Random effect models have been used.** 10) Publication bias have been discussed. 11) Potential conflict of interest has been clearly stated as several coauthors have financial interest in Biopredictive the company marketing these tests.

The Prisma flow diagram and check list were also detailed.

***AUROCs***

The secondary outcomes were the AUROC using the standard definition of liver injury and predictive values using predetermined cutoffs as defined in the validation of biomarkers in NAFLD: fibrosis defined as stage METAVIR F2,F3F4 (advanced fibrosis) and presumed when FT >0.48 and F1,F2,F3,F4 presumed when FT > 0.27; advanced steatosis defined as stage S2/S3 using NAS scoring system, >33% of parenchyma, presumed when ST >0.69 and S1, >5%-33%, presumed when ST>0.57; for NASH the definition was the NAS categories (NoNash: NAS score < 3, Possible: 3-4, Nash: score >4, categories presumed when NashTest= 0.25, 0.50 and 0.75 respectively [12]; AT accuracy for the diagnostic of Nash (NAS >4) was also assessed as observed recently [10], presumed using the threshold used for viral hepatitis AT>0.29 for METAVIR activity grade A1 and NAS >2, presumed when AT >0.17 for METAVIR A0.

A sensitivity analysis of biomarkers analysis was performed in patients with diabetes versus patients without diabetes as the risk of liver injury can be different in patients with diabetes.
