# Supplementary material for: Performance of Biomarkers FibroTest, ActiTest, SteatoTest, and NashTest in Patients with Severe Obesity: Meta Analysis of Individual Patient Data
Source: PLoS One. 2012 Mar 14;7(3):e30325. doi: 10.1371/journal.pone.0030325 (PMC3303768; doi:10.1371/journal.pone.0030325)
Supplement: Supplementary file 5 [file pone.0030325.s002.docx]

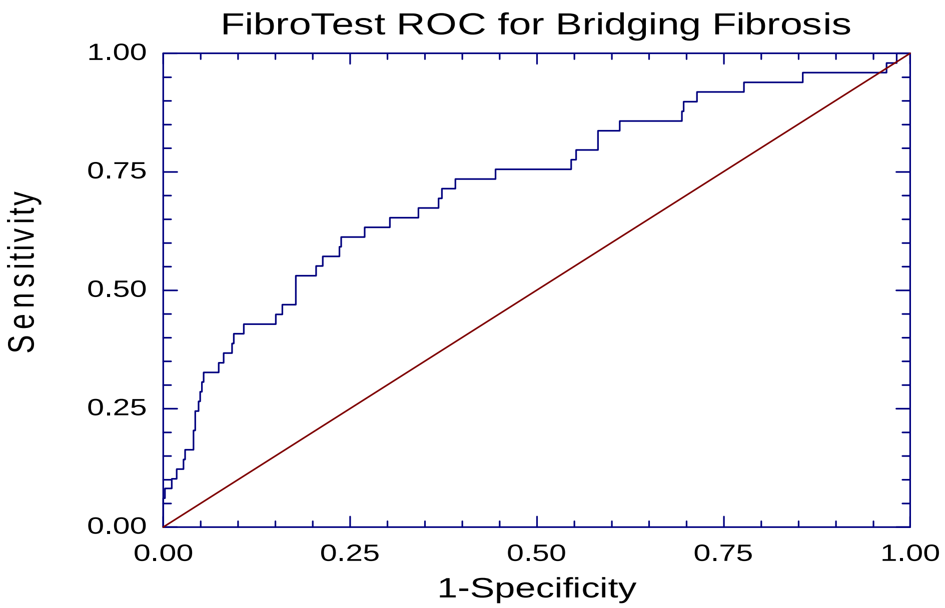
FibroTest ROC for Bridging Fibrosis
Sensitivity
1.00
0.75
0.50
0.25
0.00
0.00
0.25
0.50
0.75
1.00
1-Specificity

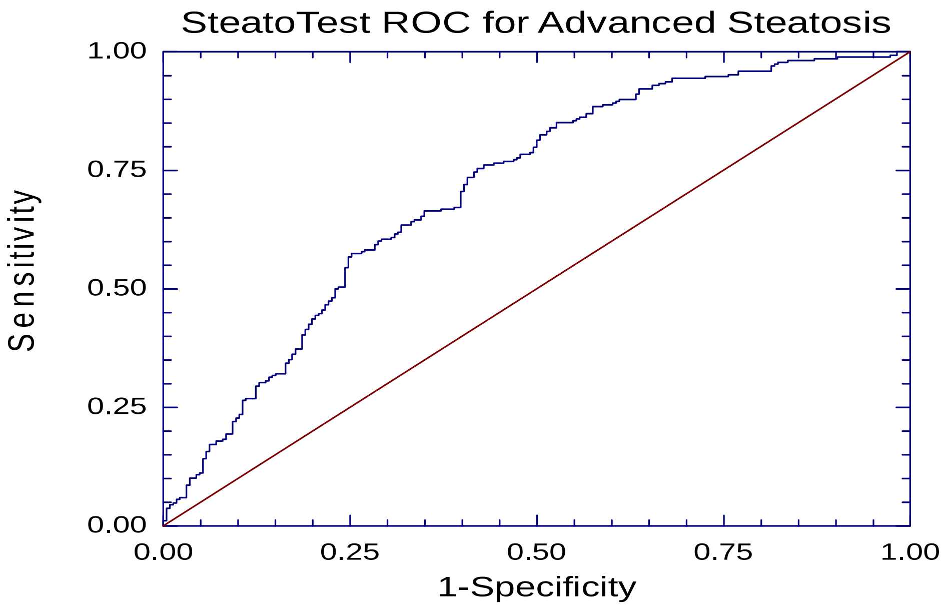
SteatoTest ROC for Advanced Steatosis
Sensitivity
1.00
0.75
0.50
0.25
0.00
0.00
0.25
0.50
0.75
1.00
1-Specificity

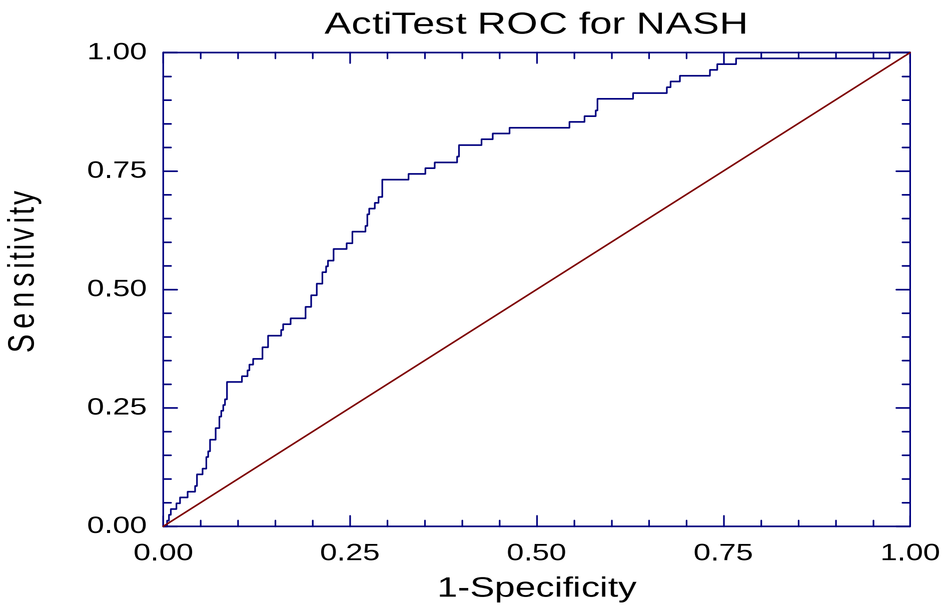

ActiTest ROC for NASH
Sensitivity
1.00
0.75
0.50
0.25
0.00
0.00
0.25
0.50
0.75
1.00
1-Specificity
